# Neurogenesis-mediated forgetting minimizes proactive interference

**DOI:** 10.1038/ncomms10838

**Published:** 2016-02-26

**Authors:** Jonathan R. Epp, Rudy Silva Mera, Stefan Köhler, Sheena A. Josselyn, Paul W. Frankland

**Affiliations:** 1 Program in Neurosciences and Mental Health, The Hospital for Sick Children, Toronto, Ontario, Canada M5G 1X8; 2 Department of Psychology, Brain and Mind Institute, University of Western Ontario, London, Ontario, Canada N6A 5B7; 3 Baycrest Centre, Rotman Research Institute, Toronto, Ontario, Canada M6A 2E1; 4 Department of Psychology, University of Toronto, Toronto, Ontario, Canada M5S 3GM; 5 Department of Physiology, University of Toronto, Toronto, Ontario, Canada M5S 1A8; 6 Institute of Medical Science, University of Toronto, Toronto, Ontario, Canada M5S 1A8

## Abstract

Established memories may interfere with the encoding of new memories, particularly when existing and new memories overlap in content. By manipulating levels of hippocampal neurogenesis, here we show that neurogenesis regulates this form of proactive interference. Increasing hippocampal neurogenesis weakens existing memories and, in doing so, facilitates the encoding of new, conflicting (but not non-conflicting) information in mice. Conversely, decreasing neurogenesis stabilizes existing memories, and impedes the encoding of new, conflicting information. These results suggest that reduced proactive interference is an adaptive benefit of neurogenesis-induced forgetting.

## Abstract (web summary)

New neurons are continuously produced throughout adulthood in the hippocampus. Here the authors provide evidence that adult hippocampal neurogenesis weakens existing memories, and facilitates the encoding of new, confliction information in mice.

## Graphical abstract

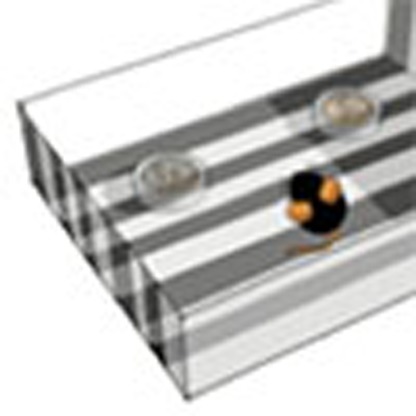

New neurons are continuously generated throughout adulthood in the subgranular zone of the hippocampus. These new neurons become synaptically integrated into hippocampal circuits over the course of weeks, and, once sufficiently mature, are thought to contribute to encoding of new hippocampus-dependent memories^1^. Consistent with this view, suppressing hippocampal neurogenesis typically impairs memory encoding^2^, whereas increasing neurogenesis facilitates this process^3^,^4^.

However, because neuronal integration necessarily remodels hippocampal circuits, computational models predict that the addition of new neurons may lead to the destabilization of existing memories, rendering them harder to access as neurogenesis unfolds^5^,^6^,^7^,^8^. Consistent with this prediction, we recently showed that increasing neurogenesis after training promoted forgetting of spatial and contextual memories in adult mice^9^.

The finding that increasing neurogenesis promotes forgetting suggests a revised view of the role of adult neurogenesis in the hippocampus, with ongoing neurogenesis impacting hippocampal memory function in two interdependent ways. First, the integration of new neurons may ‘clear out' old memories and, second, facilitate encoding of new memories^10^. Since higher levels of hippocampal neurogenesis are typically associated with improved cognitive function^11^,^12^, in what way might neurogenesis-mediated clearance improve memory function?

Here, we manipulated levels of hippocampal neurogenesis in adult mice and examined how it modulates interactions between previously acquired memories (or old information) and encoding of new information in the same behavioural task. We hypothesized that neurogenesis-mediated weakening of old memories might facilitate encoding of new, conflicting information, a process that could be characterized as a reduction of proactive interference^10^.

## Results

### Post-training exercise weakens existing spatial memories

**Figure 1:**
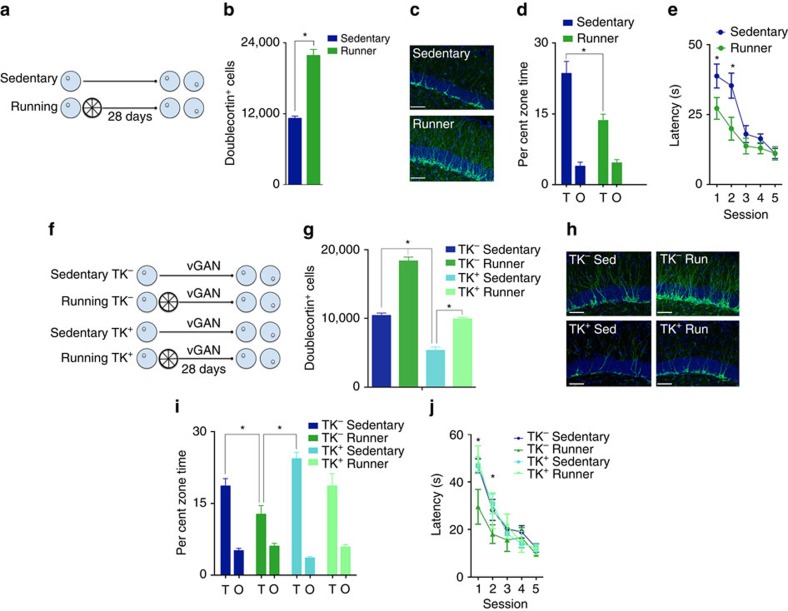
Running-induced neurogenesis promotes forgetting of previous information and consequently facilitates reversal learning. (**a**) After water maze training mice were housed conventionally (*n*=24) or given running wheel access (*n*=24) for 4 weeks before testing and reversal training. (**b**) Running increased the number of doublecortin^+^ neurons in the dentate gyrus (F_1,18_=142.03, *P*<0.001). (**c**) Examples of doublecortin^+^ immature neurons in the dentate gyrus of sedentary versus runner mice. Scale bars, 50 μm. (**d**) Running reduced retention of the platform location (Group × Zone interaction: F_1,46_=9.56, *P*<0.005), and (**e**) facilitated reversal learning (Group × Day interaction: F_1,46_=6.31, *P*<0.0001). (**f**) *TK*^*+*^ and *TK*^*−*^ mice were trained in the water maze and were then given oral vanganciclovir treatment for 4 weeks. During this period, roughly half of the mice received running wheels and the other half remained sedentary (*TK*^*−*^ sedentary *n*=14, *TK*^*+*^ sedentary *n*=17, *TK*^*−*^ runner *n*=20, *TK*^*+*^ runner *n*=23). (**g**) The running-induced increases in doublecortin^+^ cells were attenuated in *TK*^*+*^ mice (Genotype × Group interaction: F_1,43_=7.08, *P*<0.05). (**h**) Examples of doublecortin^+^ immature neurons in the dentate gyrus of sedentary versus runner mice *TK*^*−*^ and *TK*^*+*^ mice. Scale bars, 50 μm. (**i**) Post-training running induced forgetting in *TK*^*−*^ (Zone_Target_>Zone_Other_; *P*<0.05) but not in *TK*^*+*^ mice (*P*>0.05; Group × Zone interaction: F_3,74_=5.17, *P*<0.005). (**j**) Running facilitated reversal training in *TK*^*−*^ but not *TK*^*+*^ mice (Group × Day interaction: F_12,292_=1.86, *P*<0.05; T, target platform zone, O, average of three equivalent platform zones in other quadrants). Data analysis used ANOVA (**b**,**d**,**g**,**i**) and repeated-measures ANOVA (**e**,**j**). *P<0.05 by Newman–Keuls *post hoc* tests for multiple comparisons. Data shown are mean±s.e.m.

To examine how hippocampal neurogenesis might regulate proactive interference, we first trained mice in the hidden platform version of the water maze, a form of spatial learning that depends on the hippocampus^13^ (Fig. 1a). Across the course of training, mice found the platform with decreasing latency (Supplementary Fig. 1a) and, in a probe test conducted at training completion, searched selectively in the region of the pool that formerly contained the platform (Supplementary Fig. 1b). Mice were subsequently allowed continuous access to a running wheel in their home cage or housed conventionally, and then re-tested 1 month later. Consistent with previous reports^14^, running robustly increased hippocampal neurogenesis (increased numbers of proliferating cells (Supplementary Fig. 1c) and immature neurons (Fig. 1d,e)). Furthermore, running-induced increases in neurogenesis were associated with a reduction in spatial selectivity when mice were re-tested (Fig. 1b), suggesting that post-training running induced forgetting of the spatial memory^9^.

### Reversal learning is enhanced in mice that exercised

To the extent that post-training exercise weakened memory for the original platform location, we predicted that mice should be able to learn a reversal platform location more readily^10^. To test this, we shifted the platform to the opposite quadrant of the pool and continued to train the mice. Over the course of this reversal training, mice in the running group found the reversal platform more efficiently (Fig. 1c), suggesting that neurogenesis-mediated weakening of memory for the original platform location allowed more efficient encoding of a new, conflicting memory. Supporting this idea, the ease with which mice found the reversal location was inversely related to the strength of the memory for the original platform location (*r*=−0.33, *P*<0.05). However, this benefit was transient. With extended training, latencies to locate the reversal platform converged between running and sedentary groups, and, in a probe test at the completion of reversal training, both groups expressed an equivalent preference for the new platform location (Supplementary Fig. 1d).

### Reduced proactive interference depends on neurogenesis

In addition to increasing hippocampal neurogenesis, running induces a number of physiological changes^15^. To examine the importance of neurogenesis in our running effect, we next tested whether preventing the running-induced increase in neurogenesis would prevent both forgetting and subsequent facilitated reversal learning. To do this, we used mice in which levels of neurogenesis could be genetically decreased. Specifically, we trained nestin-tk (*TK*^*+*^) mice^16^ and their WT (wild-type; *TK*^*−*^) littermate controls in the water maze (Fig. 1f). Mice were subsequently allowed continuous access to a running wheel in their home cage or housed conventionally and then re-tested 4 weeks later. During this 4-week retention delay, all mice were treated with vanganciclovir via their food. In *TK*^*+*^ mice, vanganciclovir administration ablates only dividing cells expressing the *tk* gene. Accordingly, vanganciclovir treatment in *TK*^*+*^ mice limited the running-induced increase in neurogenesis to *TK*^*−*^ sedentary levels (Fig. 1i,j). Most importantly, preventing the running-induced increase in neurogenesis prevented both the forgetting (Fig. 1g) and facilitated reversal learning (Fig. 1h) normally induced by running, indicating that the increase in neurogenesis is necessary for these interrelated effects on memory.

### Decreasing neurogenesis increases proactive interference

**Figure 2:**
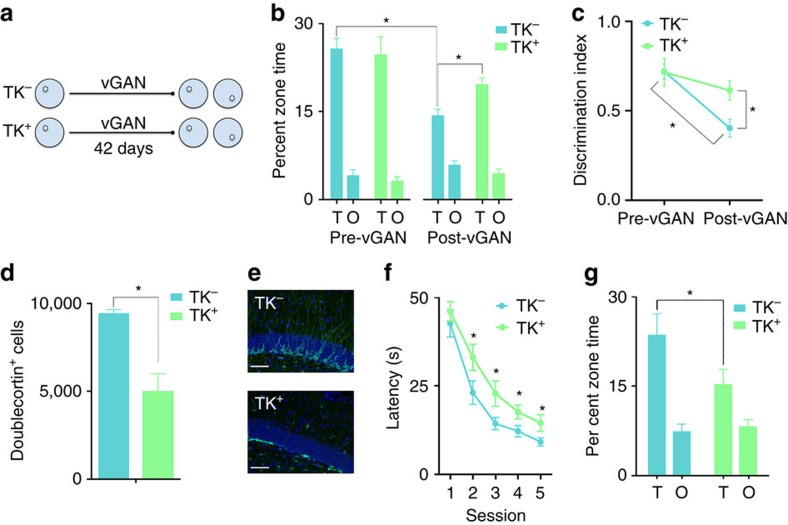
Post-training reduction of adult neurogenesis prevents forgetting of water maze platform location. (**a**) Nestin *TK*^*−*^ (*n*=12) and *TK*^*+*^ (*n*=12) mice were trained in the water maze and were then treated with vanganciclovir for 6 weeks. Spatial memory retention was probed, and then mice were trained on a reversed platform location. (**b**) Probe test performance in *TK*^*−*^ and *TK*^*+*^ mice before and after vanganciclovir treatment. Whereas *TK*^*−*^ mice searched the target zone less post-vanganciclovir (indicating forgetting of the platform location), *TK*^*+*^ searched target zone equivalently pre- versus post-vanganciclovir (indicating an absence of forgetting; Genotype × Delay interaction: F_1,22_=4.07, *P*=0.056). (**c**) Discrimination of the platform zone (compared with the opposite zone) declined in vanganciclovir-treated *TK*^*−*^ but not *TK*^*+*^ mice in the 6-week retention delay (Genotype × Delay interaction: F_1,22_=5.20, *P*<0.05). (**d**) Doublecortin^+^ neurons were reduced in *TK*^*+*^ mice (F_1,22_=47.03, *P*<0.0001). (**e**) Examples of doublecortin^+^ neurons in the dentate gyrus of *TK*^*−*^ and *TK*^*+*^ mice. Scale bars, 50 μm. (**f**) Suppression of neurogenesis impaired reversal learning in *TK*^*+*^ mice (Genotype main effect: F_1,25_=5.14, *P*<0.05). (**g**) *TK*^*+*^ mice spent less time in the target zone compared with *TK*^*−*^ mice (Genotype × Zone interaction: F_1,22_=4.42, *P*<0.05). Data analysis used ANOVA (**b**,**c**,**d**,**g**) and repeated-measures ANOVA (**f**). *P<0.05 by Newman–Keuls *post hoc* tests for multiple comparisons. Data shown are mean±s.e.m.

To the extent that post-training increases in neurogenesis induce forgetting, we predicted that post-training reductions in neurogenesis would have the opposite effect and stabilize hippocampus-dependent memories^10^. In our initial experiments we did not observe reliable forgetting in control mice when we used a 4-week retention delay. Therefore, to increase the probability of observing forgetting in control mice, we extended the retention delay to 6 weeks in this experiment (Fig. 2a). Using this delay we observed forgetting, with *TK*^*−*^ mice searching less selectively in the probe test conducted at the end of the retention delay (Fig. 2b,c). Critically, genetic suppression of hippocampal neurogenesis (Fig. 2f,g) in the post-training window attenuated this forgetting, with vanganciclovir-treated *TK*^*+*^ mice searching more selectively compared with vanganciclovir-treated *TK*^*−*^ mice (Fig. 2b,c). Consistent with this preserved spatial selectivity, *TK*^*+*^ mice were slower at finding the platform when it was subsequently moved to the opposite quadrant (Fig. 2d,e), indicating that relative preservation of the original memory interfered with subsequent learning of a conflicting platform location.

### Increasing neurogenesis decreases proactive interference

**Figure 3:**
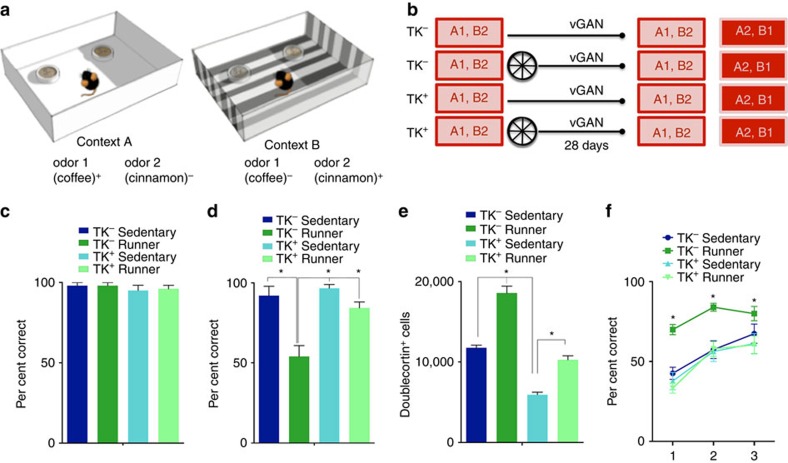
Post-training induction of neurogenesis promotes forgetting of an odour–context paired-associates memory and consequently facilitates reversal learning. (**a**) Mice were presented with two different cups of scented bedding in two different contexts and were trained to dig in the bedding to receive a food reward. (**b**) *TK*^*−*^ and *TK*^*+*^ mice were trained on odour–context pairings A1 and B2. Subsequently the mice either ran (*TK*^*−*^ *n*=6, *TK*^*+*^ *n*=9) for 4 weeks or remained sedentary (*TK*^*−*^ *n*=5, *TK*^*+*^ *n*=8). During this period all mice received oral vanganciclovir treatment. After 4 weeks the mice were tested on the original odour–context pairings and were then subjected to reversal learning (that is, A2 and B1). (**c**) At the end of training all groups performed >80% correct (*F*_1,24_=0.03, *P*>0.86). (**d**) Post-training running induced forgetting in *TK*^*−*^ but not in *TK*^*+*^ mice (*P*>0.05; Group × Genotype interaction: F_1,24_=8.66, *P*<0.005). (**e**) The running-induced increase in doublecortin^+^ cells was attenuated in *TK*^*+*^ mice (Genotype × Group interaction: F_1,16_=5.29, *P*< 0.05). (**f**) Running facilitated reversal training in *TK*^*−*^ but not *TK*^*+*^ mice (Group × Genotype interaction: F_1,24_=8.66, *P*<0.005). Data analysis used ANOVA (**c**,**d**,**e**) and repeated-measures ANOVA (**f**). *P<0.05 by Newman–Keuls *post hoc* tests for multiple comparisons. Data shown are mean±s.e.m.

We next evaluated whether this pattern of results would generalize to other forms of hippocampus-dependent learning. To do this we trained *TK*^*−*^ and *TK*^*+*^ mice in a hippocampus-dependent^17^,^18^ odour–context paired associates task. In this task, mice were trained in two contexts, each containing two containers scented with cinnamon or coffee. During training, digging in one of these scented containers (for example, coffee) was reinforced in context A, whereas digging in the other scented container (for example, cinnamon) was reinforced in context B (Fig. 3a,b; Supplementary Table 1). All mice reached stable, high levels of performance (that is, correct on >80% of trials) by the completion of training (Fig. 3c). Mice were subsequently given continuous access to a running wheel in their home cage or housed conventionally, and then re-tested 4 weeks later. During this period, all mice were treated with vanganciclovir via their food. In the test, *TK*^*−*^ mice that exercised after initial training showed inferior memory for the paired associates compared with sedentary controls. In contrast, exercise did not impair memory for the original paired associates in *TK*^*+*^ mice (Fig. 3d). Consistent with the water maze results, these data indicate that post-training exercise weakens an established hippocampus-dependent memory. Unaffected paired associate memory in the *TK*^*+*^ mice with reduced hippocampal neurogenesis (Fig. 3e) suggests that these forgetting effects are mediated via a neurogenic mechanism.

Mice were subsequently retrained with the original context/odour contingencies reversed. Acquisition of these reversed paired associates was facilitated only in the *TK*^*−*^ mice that had exercised following learning the original paired associates (Fig. 3f). Consistent with the water maze results, these data indicate that post-training exercise weakens an established hippocampus-dependent memory and, in turn, this facilitates acquisition of new paired associates that conflict with the original learning. Supporting this idea, the ease with which mice learned the reversed paired associates was inversely related to the strength of the memory for the original paired associates (*r*=−0.51, *P*<0.01). Importantly, attenuating running-induced increases in hippocampal neurogenesis prevented both the forgetting and facilitated reversal learning in *TK*^*+*^ mice, indicating that hippocampal neurogenesis mediates these interrelated effects on memory.

### Reduced interference is specific to new conflicting memories

In the above experiments, neurogenesis-induced forgetting might facilitate reversal learning by reducing conflict between the original learning and the new learning. Alternately, improved reversal learning might reflect a more general enhancement of learning following elevation of hippocampal neurogenesis (independent of degree of conflict)^3^,^4^ (see also Supplementary Fig. 2). To distinguish between these alternatives we modified the odour–context paired associates protocol so that the degree of conflict between old and new paired associates could be systematically manipulated. In this case, mice were trained as previously and again reached stable, high levels of performance (that is, correct on >80% of trials) by the completion of training (Supplementary Fig. 3a). Mice were subsequently given continuous access to a running wheel in their home cage or housed conventionally, and then re-tested 4 weeks later. Running increased neurogenesis (Supplementary Fig. 3b), and weakened memory for the original paired associates (tested using diluted odours; Supplementary Fig. 3c). As expected, this indicates that running weakened the memory for the original paired associates, impeding pattern completion when cue salience was diminished.

**Figure 4:**
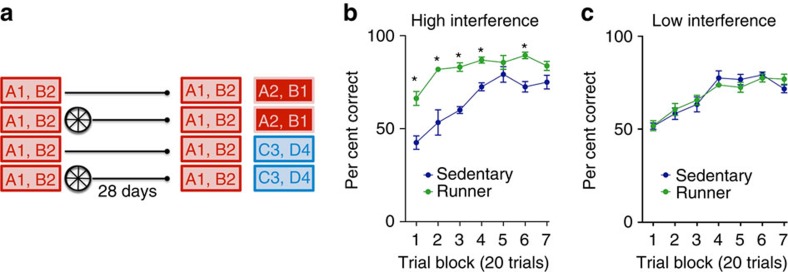
High- but not low-interference reversal learning is facilitated by running-induced neurogenesis. (**a**) Mice were trained on odour–context pairings A1 and B2. Subsequently the mice either ran (*n*=16) for 4 weeks or remained sedentary (*n*=12). After 4 weeks the mice were tested on the original odour–context pairings and were then subjected to either reversal learning (that is, A2 and B1 (high interference)) or were trained on two new odour–context pairings (that is, C3 and D4 (low interference)). (**b**,**c**) Runners learned faster than sedentary mice in the high interference (Group main effect: F_1,72_=94.79, *P*<0.0001; Group × Session interaction: F_6,72_=3.40, *P*<0.01) but not low-interference (Group main effect: F_1,72_=0.00, *P*>0.05) condition. Data analysis used repeated-measures ANOVA (**b**,**c**). *P<0.05 by Newman–Keuls *post hoc* tests for multiple comparisons. Data shown are mean±s.e.m.

Mice were subsequently trained on new paired associates that were either in conflict (that is, reversal of original context/odour contingencies) or not (that is, new context/odour pairs) with the original learning (Fig. 4a). Running-induced increases in hippocampal neurogenesis facilitated the acquisition of new paired associates, but only in the ‘high conflict' condition (Fig. 4b). The absence of effects on learning the ‘low conflict' paired associates (Fig. 4c) argues against the possibility that elevated neurogenesis has a general facilitatory effect on learning, at least under these conditions. Rather, facilitation was only observed under conditions where there was conflict between previously stored memories and new learning. Consistent with this interpretation, in separate experiments we confirmed that exercise does not facilitate acquisition of paired associates (or subsequent reversed paired associates) when it occurs before training (Supplementary Figs 4,5).

## Discussion

Previously we showed that elevated levels of neurogenesis promote forgetting of established memories^9^. Here we hypothesized that one benefit of this neurogenesis-mediated forgetting might be more efficient encoding of new, conflicting information. Using two distinct hippocampus-dependent tasks, we provide support for this idea: Increasing hippocampal neurogenesis after initial learning reduced this form of proactive interference, whereas decreasing hippocampal neurogenesis after learning increased this form of proactive interference. Our results suggest that heightened levels of hippocampal neurogenesis might allow animals to react more rapidly to changing contingencies in otherwise stable environments.

Various roles have previously been assigned to the hippocampus with respect to mitigating interference (for example, see refs 19, 20, 21, 22, 23). In particular, some earlier studies investigated the relationship between levels of hippocampal neurogenesis and memory interference (as well as the closely related concept of cognitive flexibility)^24^,^25^,^26^,^27^,^28^,^29^. For example, in one study hippocampal neurogenesis levels were experimentally suppressed in mice. Mice were subsequently trained to find a hidden platform in a fixed location in the water maze. In a second phase of training, the platform was moved to a different location in the pool (that is, reversal learning). Reversal, but not initial, learning was impaired in mice in which hippocampal neurogenesis was reduced^25^. However, in this and related studies^24^,^26^,^27^,^28^,^29^, manipulation of hippocampal neurogenesis levels occurred before initial learning. Therefore, it is unclear whether improved reversal learning is related to differences in initial learning or differences that emerge at the reversal stage. By restricting our manipulations of neurogenesis to the post-learning period, here we were able to show that decreased proactive interference is directly related to neurogenesis-mediated forgetting of the original learning. This effect may ultimately contribute to increases in cognitive flexibility.

A perhaps unexpected observation was that increasing hippocampal neurogenesis did not generally improve learning. Rather, in the paired associate paradigm, facilitated acquisition was only observed in the high interference condition where paired associates were reversed. In contrast, in the low interference condition, as well as in related control experiments, increasing hippocampal neurogenesis did not facilitate paired associate learning. This contrasts with some previous studies where increasing neurogenesis before training facilitated memory formation (for example, see refs 3, 4, 30). Indeed, in the current study we also observed improved acquisition in the water maze when running occurred before training (Supplementary Fig. 2). However, it is worth noting that these types of improvements are not universally observed (for example, refs 9, 31, 32). There may be a number of reasons why this is the case—ranging from differences in amount and duration of training, task difficulty, as well as the timing of the increase in neurogenesis with respect to the initiation of training.

Finally, what is forgotten? Experiences are thought to be represented in parallel hippocampal–cortical networks. According to one prominent model^33^, hippocampal representations contain high levels of contextual detail, whereas cortical representations are more schematic in nature and contain fewer contextual details. Therefore, continuous integration of new neurons into hippocampal circuits would be predicted to degrade this contextually dense hippocampal representation. The observed facilitation of reversal learning following elevation of hippocampal neurogenesis in the water maze and paired-associates task is consistent with such an account. In particular, in the paired-associates task, uncoupling odour-reward relationships from their contexts enables more efficient reversal learning.

## Methods

### Mice

Two strains of mice were used in these experiments. First, we used WT mice that were derived from a cross between *129Svev* and *C57Bl/6N* mice (Taconic). Second, to suppress adult neurogenesis we used transgenic mice expressing a modified HSV-*tk* gene under control of the nestin promoter (*TK*^*+*^ mice)^16^,^34^. These mice were maintained on a *C57Bl/6N* background (>8 crosses), and experimental mice were generated by crossing *TK*^*+*^ mice with *129Svev* WT mice. In all experiments, we used both male and female mice. Breeding was conducted in the Hospital for Sick Children animal facility. Mice were housed in standard laboratory conditions with 3–5 mice per cage and had free access to food and water. Rooms were maintained on a 12 h light/dark cycle and behavioural testing occurred during the light phase of the cycle. Experiments were conducted in accordance with the Canadian Council on Animal Care guidelines and the protocols were approved by the animal care committee at the Hospital for Sick Children.

### Manipulation of neurogenesis

To increase adult neurogenesis, mice were given continuous access to a running wheel (Fast-Trac running wheels, Bioserv) in their home cages^9^. When the wheel was initially introduced to the cage each mouse was gently placed on the wheel to facilitate running wheel use. In a subset of cages, wheel revolutions were continuously recorded to track amount of running. Each mouse ran an average of 4.7 km (±0.53 km) per day during the running period.

To decrease adult neurogenesis *TK*^*+*^ mice and non-transgenic littermate (*TK*^*−*^) control mice were administered vanganciclovir orally. Vanganciclovir was mixed into powdered chow (0.08%, ∼70 mg kg^−1^ daily)^2^, and mice had *ad libitum* access to the chow in their home cage.

### Water maze apparatus and general procedures

Before water maze training, mice were handled for 5 min per day over 5 consecutive days. Water maze training was conducted in a pool 120 cm in diameter filled to within 10 cm of the rim. The water was made opaque with non-toxic white paint. A hidden platform (10 cm diameter) was submerged in the centre of the northwest quadrant of the pool. Training consisted of five daily sessions each comprising six trials with a maximum trial length of 60 s. At the start of each trial, the mouse was lowered into the water in one of four start positions (located in the north, south, east and west, respectively), and the order of these start positions was varied pseudorandomly across trials. At the end of each trial the mouse remained on the platform for an additional 15 s. If the mouse failed to locate the platform within 60 s it was gently guided to the platform by the experimenter at the completion of the trial.

To assess spatial preference, 24 h following the completion of training, mice were given a 60 s probe trial. In this trial the platform was removed from the pool, and a southeast start position was used. The amount of time spent in a zone centred on the former platform location (radius 15 cm) was compared with the time spent in equivalent zones centred in the other three quadrants. The zone represents 6.7% of total pool-surface area.

Reversal training used identical procedures except that the platform was relocated to the middle of the southeast quadrant. Mice were trained for 5 days, and each daily training session comprised three trials. Twenty-four hours following reversal training, spatial preference was assessed in a probe test. In this reversal probe test we additionally computed a discrimination score (time spent in the reversal platform zone/time spent in the original platform zone).

### Water maze experiment 1

Does post-training exercise reduce retention and facilitate reversal learning? WT mice were trained in the water maze for 5 days. Twenty-four hours after the completion of training, mice were given a probe test, and then either housed with a running wheel (runner group; *n*=24) or without a running wheel (sedentary group; *n*=24). Four weeks later, retention was evaluated in a probe test and, starting the following day, mice were trained to locate the platform in the opposite quadrant of the pool for 5 days. Twenty-four hours after the completion of this reversal training, a probe trial was conducted to assess memory for the new platform location.

### Water maze experiment 2

Are exercise-induced changes in retention and reversal learning dependent upon neurogenesis? *TK*^*+*^ and their WT littermate control mice were trained in the water maze for 5 days, and spatial memory was assessed 24 h following the completion of training. Starting the next day, all mice were adminsitered vanganciclovir via chow in their home cage for a 4-week retention period. During this period, some mice had access to running wheels (WT runner, *n*=20; *TK*^*+*^ runner, *n*=23), while others were housed conventionally (WT sedentary, *n*=14; *TK*^*+*^ sedentary, *n*=17). Spatial memory was tested in a probe test, and then, the following day, reversal learning was initiated. Twenty-four hours following the completion of reversal training, mice were presented with a second probe test.

### Water maze experiment 3

Does post-training suppression of neurogenesis increase retention and retard reversal learning? *TK*^*+*^ (*n*=12) and their WT littermate control (*n*=12) mice were trained in the water maze for 5 days, and spatial memory was assessed 24 h following the completion of training. Starting the next day, all mice were administered vanganciclovir via chow in their home cage for a 6-week retention period. Spatial memory was then tested in a probe test. One day later, reversal learning was initiated. Twenty-four hours following the completion of reversal training, spatial preference was assessed in a second probe test.

### Water maze experiment 4

Does pre-training exercise facilitate acquisition of spatial learning? Starting at 8 weeks of age, WT mice were given access to running wheels (*n*=15) or remained sedentary (*n*=16) for a period of 4 weeks. Mice were trained in the water maze for 5 days, and spatial memory was assessed in a probe test 24 h after the completion of training.

### Paired-associates task

We trained *TK*^*+*^ and their WT littermate control mice on an odour/context paired-associates task^17^,^18^ in which the mice were required to dig in a container of bedding scented with 1% spice (for example, coffee or cinnamon) to receive a reward. Odour pairs were presented in two different contexts. In context A odour 1 was rewarded while in context B odour 2 was rewarded. The experimental procedures involved the following phases in sequence.

### Habituation and shaping

Mice were first habituated to the testing apparatus for 2 days. Testing was conducted in empty mouse cages containing two 60 × 15 mm petri dishes filled with corncob bedding. During the habituation sessions, the reward, chocolate-flavoured sprinkles (10 mg, McCormick, Canada), was available on top of the bedding. Next, the mice were shaped to dig in unscented bedding to receive the reward by first partially, and then completely, burying the sprinkles in the bedding. Once the mice could consistently dig to retrieve a sprinkle, the reward was no longer buried in the bedding. Instead, a single sprinkle was delivered on top of the bedding immediately after the mouse dug in the bedding. Shaping took five sessions.

### Simple discrimination

Two different cups were scented with vegetable seasoning or tumeric, and mice were rewarded for digging only in the vegetable scented bedding. Each trial ended after the mice made their first choice. Simple discrimination training typically took 5 sessions to reach a criterion of 80% correct.

### Paired-associates training

We next introduced two new contexts and two new odours. Context A was a white Plexiglass box and context B was a black and white striped box (context B). The dimensions of both contexts were the same (32 × 18 × 14 cm). The new odours were coffee (odour 1) and cinnamon (odour 2). In context A, odour 1 was rewarded but in context B odour 2 was rewarded. Mice were initially trained with 10 trials per day in a single context. Contexts alternated across days. Once a mouse reached 70% correct in both contexts we began presenting the mice with five trials of each context per session in a pseudorandom pattern (A-B-A-AB-A-B-B-A-B). Mice were trained until they reached a criterion of at least 80% correct in 3 consecutive sessions. Once this criterion had been reached, mice were housed conventionally (*n*=13) or given free home cage access to a running wheels (*n*=15) for the next 4 weeks. All mice were fed a powdered-food diet containing vanganciclovir during the 4-week period.

### Testing

Memory for the paired associates was tested following the 4-week retention delay. This session comprised 10 trials, with 5 trials in each context.

### Reversal training

Next, mice were trained on a reversal learning procedure. In this task the original odour-context pairings were reversed. In context A, odour 2 (for example, cinnamon) was now rewarded and in context B odour 1 (for example, coffee) was rewarded. Mice were given 20 trials per session with 10 trials of each context per session for 3 days.

### Interference training

In a separate experiment we assessed the impact of varying degrees of interference during the reversal learning period in WT mice. Memory for the paired associates was tested in six daily sessions following the 4-week retention delay. These sessions comprised 10 trials, with 5 trials in each context. For sessions 1–3 we used the original (training) odour concentrations (100% odour condition). For sessions 4–6 we used 50% (that is, 0.5% spices in corncob bedding) of the original odour concentration to evaluate retention with weaker retrieval cues. Following testing WT mice were assigned to either high interference (sedentary, *n*=6; runner, *n*=8) or low interference (sedentary, *n*=6; runner, *n*=8) training.

### High-interference training

In the high-interference condition, the original odour–context pairings used during training were reversed. In context A, odour 2 (for example, cinnamon) was now rewarded and in context B odour 1 (for example, coffee) was rewarded. Mice were given 20 trials per session with 10 trials of each context per session for 7 days.

### Low-interference training

In the low-interference condition, two new odours and two new contexts were presented. Context C (polka dot wallpaper) and context D (silver metal box) were paired with odours 3 (ginger) and 4 (oregano). Mice were given 20 intermixed trials per session for 7 days.

### Pre-training running

In separate experiments mice were habituated, shaped and trained in simple discrimination training. They were then housed conventionally (*n*=16) or given running wheel access (*n*=16) for 4 weeks before beginning the paired associate training for 12 days. Two different odour–context pairings were used for training. In the first condition the mice were trained with the normal odour–context pairings (contexts A and B and odours 1 and 2, *n*=8 per group). In the second condition mice were trained with the odour–context pairings that were otherwise used for the low-interference condition (contexts C and D and odours 3 and 4, *n*=8 per group). In a third condition mice were trained on the normal odour–context pairings (A1, B2) for a period of 6 days and were then trained on the reversal condition for an additional 6 days (A2, B1).

### Histology

Mice were deeply anaesthetised with 4% chloral hydrate and were perfused transcardially with 40 ml of 0.1 M PBS followed by 40 ml of 4% buffered formaldehyde. Brains were extracted and post-fixed in 4% formaldehyde for 24 h and were then cryoprotected in 30% sucrose for 72 h. Sections of 50 μm were cut on a cryostat, and free-floating sections were stored in 0.02% sodium azide in 0.1 M PBS. Sections were incubated for 48 h in goat anti-doublecortin primary antibody (Santa Cruz, SC-8066) diluted 1:200 in 0.3% Triton-X and 3% normal serum. The sections were then incubated for 24 h in Alexa488 Donkey anti-goat secondary antibody diluted 1:500 in PBS. The tissue was counterstained with DAPI and coverslipped with PVA-DABCO. Doublecortin was quantified using an epifluorescent microscope and × 40 objective. Cells were counted through the anterior–posterior extent of the dentate gyrus using a 1/6 sampling fraction.

### Statistical analysis

Data were analysed using ANOVA and repeated-measures ANOVA with an *α*=0.05. Where applicable, Newman–Keuls *post hoc* comparisons were computed. All analyses were carried out using the software package Statistica (Statsoft, version 10).

## Additional information

**How to cite this article:** Epp, J. R. *et al.* Neurogenesis-mediated forgetting minimizes proactive interference. *Nat. Commun.* 7:10838 doi: 10.1038/ncomms10838 (2016).

## Supplementary Material

Supplementary Information Supplementary Figures 1-5 and Supplementary Table 1
