# Supplementary material for: Neurogenesis-mediated forgetting minimizes proactive interference
Source: Nat Commun. 2016 Feb 26;7:10838. doi: 10.1038/ncomms10838 (PMC4773435; doi:10.1038/ncomms10838)
Supplement: Supplementary Information — Supplementary Figures 1-5 and Supplementary Table 1 [file ncomms10838-s1.pdf]

Supplementery Information

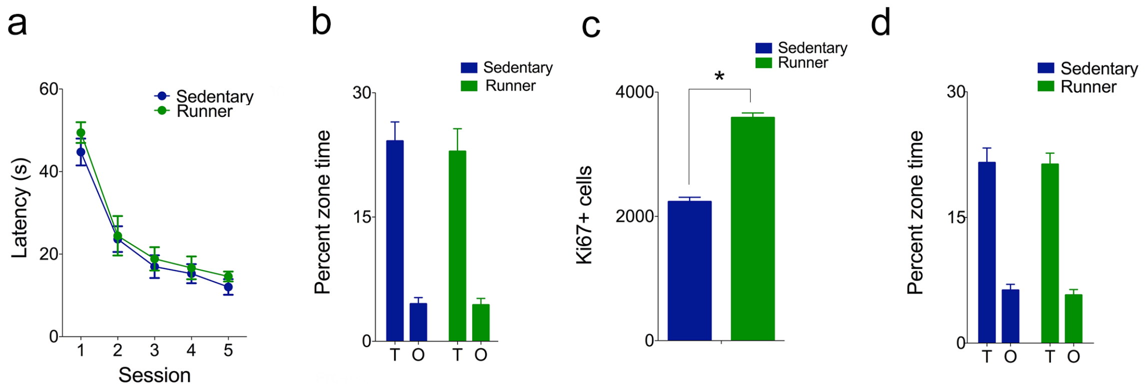

Supplementary Figure 1. **a**. Latency to locate platform in water maze training did not differ in mice assigned to running vs. sedentary group [Main effect of Day: $F_{4,184} = 139.69$, $p < 0.00001$; Group and Group × Day interaction, *F*s < 1]. **b**. At the completion of training both runner (n=24) and sedentary mice (n=24) searched selectively in the zone of the pool that formerly contained the platform [Main effect of Zone: $F_{1,46} = 216.38$, $p < 0.00001$; Group and Group × Zone interaction, *F*s < 1]. **c**. Numbers of proliferating (i.e., $Ki67^{+}$) cells were elevated following running [$F_{1,12} = 24.79$, $p < 0.001$]. **d**. At the completion of reversal training both runner and sedentary mice searched selectively in the zone of the pool where the reversal platform had been located [Main effect of Zone: $F_{1,46} = 141.05$, $p < 0.00001$; Group and Group × Zone interaction, *F*s < 1]. Data analysis used ANOVA (panels b,c,d) and repeated measures ANOVA (panel a). Data shown are mean ± SEM.

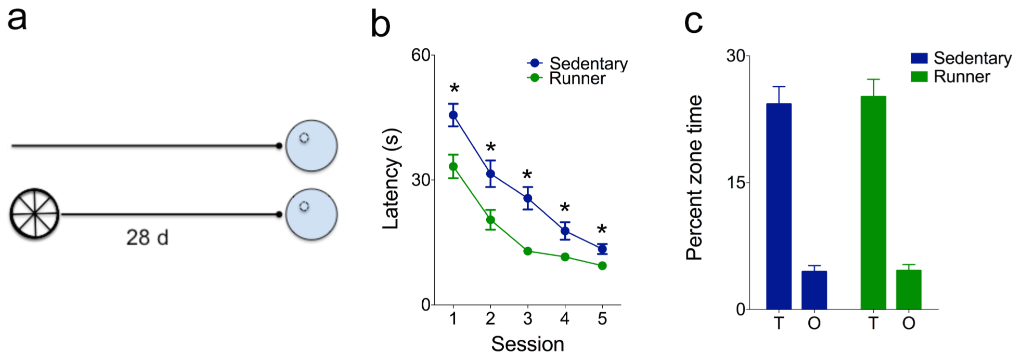

Supplementary Figure 2. **a.** Mice were given access to a running wheel (n=15) or remained sedentary (n=16) for 4 weeks prior to water maze training. **b.** Mice that ran before training located the platform more efficiently (Main effects of Day [$F_{4,120} = 61.46$, $p < 0.00001$] and Group [$F_{4,120} = 29.75$, $p < 0.00001$]). **C**. At the completion of training, both runner and sedentary mice searched selectively in the zone of the pool that formerly contained the platform [Main effect of Zone: $F_{1,30} = 205.24$, $p < 0.00001$; Group and Group × Zone interaction, *F*s < 1]. Data analysis used ANOVA (panel c) and repeated measures ANOVA (panel b). *$p < 0.05$ by Newman-Keuls post hoc tests for multiple comparisons. Data shown are mean ± SEM.

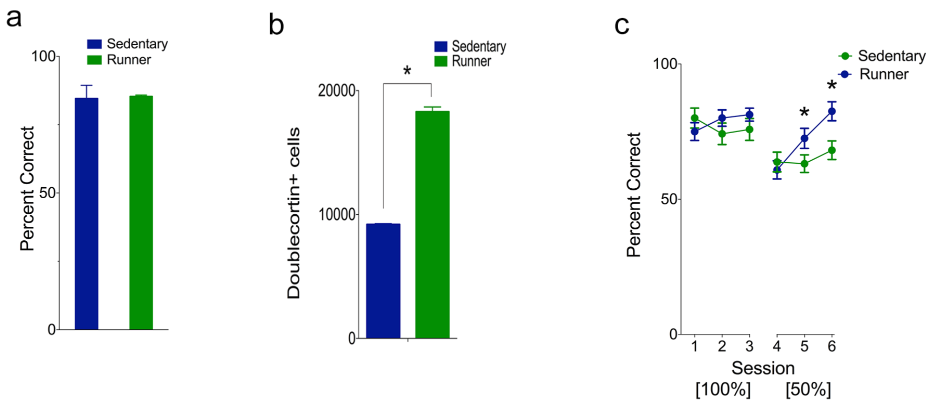

Supplementary Figure 3. **a**. Mice (n=12 sedentary, n=16 runner) reached a stable high level of performance at the end of the training period ($F_{1,26}$ = 0.05, p > 0.05). **b**. Running increased the number of immature neurons in the dentate gyrus [$F_{1,12}$ = 48.08, p < 0.00001]. **c.** After 4 weeks of running there was no significant difference in retention of the odor context pairings (test sessions 1-3). However, when the odor concentration was decreased performance was reduced in mice that ran after training [Group × Session interaction: $F_{5,130}$ = 2.87, p < 0.05]. Data analysis used ANOVA (panel a,b) and repeated measures ANOVA (panel c). *p < 0.05 by Newman-Keuls post hoc tests for multiple comparisons. Data shown are mean ± SEM.

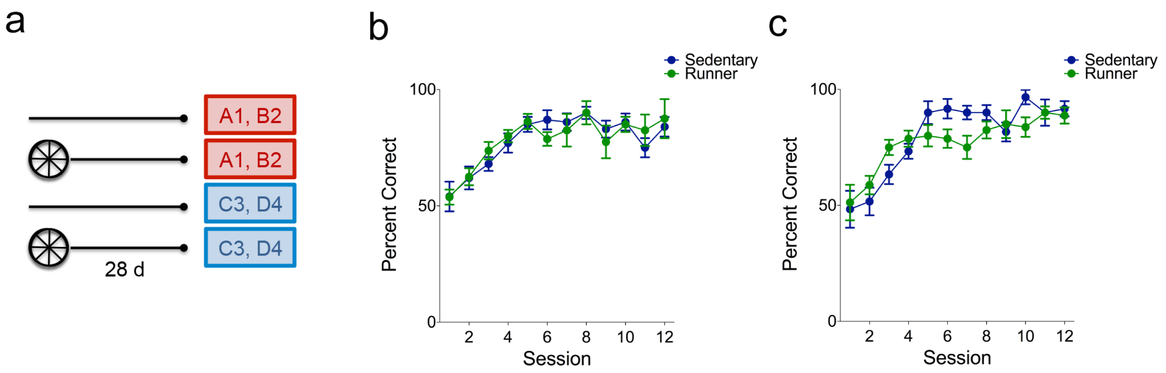

Supplementary Figure 4. **a**. Mice had access to a running wheel (n=8) for 4 weeks or were housed conventionally (n=8), and then were trained in the odor-context paired associate task (either A1-B2 or C3-D4). **b-c**. Acquisition was similar in runner (n=8) and sedentary (n=8) mice for both odor-context pairs (A1-B2, main effect of session only, $F_{11,154} = 10.73$, $p < 0.00001$; C3-D4, main effect of session only, $F_{11,154} = 16.29$, $p < 0.00001$). Data analysis used repeated measures ANOVA (panels b,c). Data shown are mean ± SEM.

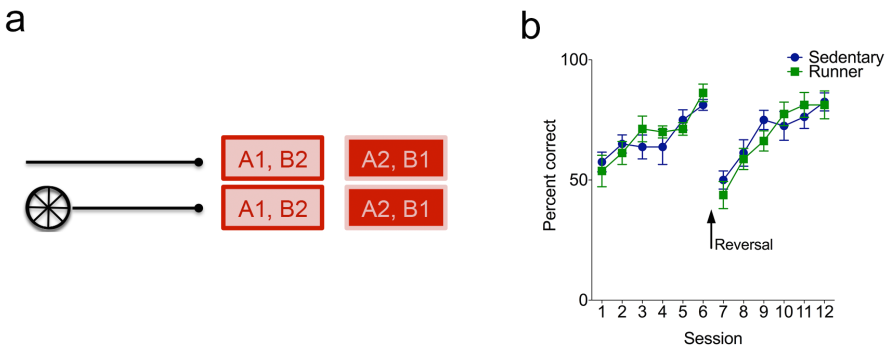

Supplementary Figure 5. **a**. Mice had access to a running wheel for 4 weeks (n=8) or were housed without a wheel (n=8). The mice were then trained in the odor-context paired associates task. After acquiring the A1,B2 pairing the mice were trained in a reversal condition (A2,B1). **b.** There was no significant difference in either the acquisition of the initial odor context pairing or the reversal pairings (Main effect of Session only: $F_{5,140} = 27.92$, $p < 0.00001$; Group × Session x Phase interaction: $F_{5,140} = 0.98$, $p = 0.43$). Data analysis used repeated measures ANOVA (panel b). Data shown are mean ± SEM.

Supplementary Table 1. Odor-context pairings in the training, high interference and low interference conditions.

	Training		High interference		Low interference	
	Context A	Context B	Context A	Context B	Context C	Context D
#1 Coffee	S+	S-	S-	S+		
#2 Cinnamon	S-	S+	S+	S-		
#3 Ginger					S+	S-
#4 Oregano					S-	S+
